# Sterol composition of shellfish species commonly consumed in the United States

**DOI:** 10.3402/fnr.v56i0.18931

**Published:** 2012-10-29

**Authors:** Katherine M. Phillips, David M. Ruggio, Jacob Exler, Kristine Y. Patterson

**Affiliations:** 1 Biochemistry Department, Virginia Tech, Blacksburg, VA; 2 USDA Agricultural Research Service, Nutrient Data Laboratory, Beltsville, MD

**Keywords:** phytosterols, salmon, crustaceans, poriferasterol, 22,23-dihydrostigmasterol, 22-dihydrobrassicasterol, desmosterol, dihydrocholesterol, 7-dehydrocholesterol, occelasterol

## Abstract

**Background:**

Shellfish can be a component of a healthy diet due to a low fat and high protein content, but the cholesterol content of some species is often cited as a reason to limit their consumption. Data on levels of non-cholesterol sterols in commonly consumed species are lacking.

**Objective:**

Shellfish were sampled and analyzed to update sterol data in the United States Department of Agriculture (USDA) National Nutrient Database for Standard Reference.

**Design:**

Using a nationwide sampling plan, raw shrimp and sea scallops, canned clams, and steamed oysters, blue crab, and lobster were sampled from 12 statistically selected supermarkets across the United States in 2007–08. For each species, four composites were analyzed, each comprised of samples from three locations; shrimp and scallops from six single locations were also analyzed separately. Using validated analytical methodology, 14 sterols were determined in total lipid extracts after saponification and derivatization to trimethylsilyethers, using gas chromatography for quantitation and mass spectrometry for confirmation of components.

**Results:**

Crab, lobster, and shrimp contained significant cholesterol (96.2–27 mg/100 g); scallops and clams had the lowest concentrations (23.4–30.1 mg/100 g). Variability in cholesterol among single-location samples of shrimp was low. The major sterols in the mollusks were brassicasterol (12.6–45.6 mg/100 g) and 24-methylenecholesterol (16.7–41.9 mg/100 g), with the highest concentrations in oysters. Total non-cholesterol sterols were 46.5–75.6 mg/100 g in five single-location scallops samples, but 107 mg/100 g in the sixth, with cholesterol also higher in that sample. Other prominent non-cholesterol sterols in mollusks were 22-dehydrocholesterol, isofucosterol, clionasterol, campesterol, and 24-norcholesta-5,22-diene-3β-ol (4–21 mg/100 g).

**Conclusions:**

The presence of a wide range of sterols, including isomeric forms, in shellfish makes the analysis and quantitation of sterols in marine species more complex than in animal and plant tissues. The detailed sterol composition reported herein provides data that may be useful in research on the impact of shellfish consumption on dietary risk factors.

Shellfish can be a component of a healthy diet due to a low fat and high protein content. Per capita, intake of the 10 most highly consumed shellfish species in the US in 2010 was 15.8 lb, with shrimp (4.0 lb), crab (0.573 lb), and clams (0.341 lb) collectively comprising 31% of that total (1). However, the cholesterol content of some species is sometimes cited as a limitation to their consumption.

The reported effects of shellfish (mollusks and crustaceans) on blood cholesterol levels and cardiovascular disease (CVD) risk factors have been variable. For example, Connor and Lin (2) found lobster, crab, and shrimp but not clams, oysters, and scallops to be mildly hypercholesterolemic in individuals with normal blood cholesterol levels. In a second study with a diet containing clams, oysters and scallops, the cholesterol of the two normal men was unaffected, but the blood cholesterol in a hypercholesterolemic woman increased. Childs et al. (3) found that diets containing oysters and clams versus chicken or crab but with equivalent omega-3 fatty acid, cholesterol, and energy contents inhibited cholesterol absorption and that compared with the chicken, the oyster and clam diet increased the HDL_2_-/HDL_3_-cholesterol ratio in normocholesterolemic patients. The role of diet in modulating blood cholesterol levels and CVD risk is not straightforward. Other dietary components, lifestyle, and differences in human genotypes may play a role, interact, and have different effects in different individuals. Teupser et al. (4) reported on genetic influences that impact the effect of dietary sterols among individuals. The beneficial effects of cholesterol and phytosterol intake on blood cholesterol levels and CVD risk factors have been reviewed (5–7).

Accurate food composition data, along with estimates of variability in the food supply, are needed to support epidemiological studies. Crustaceans contain cholesterol at relatively high levels when compared to muscle meats (e.g., >120 mg/100 g in steamed lobster vs. 82 mg/100 g in 90% cooked lean ground beef (8). Crustaceans and mollusks also contain a variety of other sterols, some unique to marine species (9–13). These sterols are not represented in food composition databases, which only contain values for cholesterol and, in some cases, selected phytosterols. Additionally, existing literature reports on shellfish sterol composition were conducted for the purpose of comparative biochemistry and physiology and many involved single species and/or a limited number of samples. Therefore, these data, on a statistical basis, are not necessarily representative of the composition of these species in the retail market. Environmental and dietary factors can affect the sterol content of shellfish harvested from different natural locations or farm-raised (14–17). Thus, a representative sampling plan that accounts for natural variability in these species as they occur in the food supply is important in the context of food composition databases that are used to estimate dietary intake in a population. While Tsape et al. (18) reported on sterols in selected crustacean species sampled in Greece, there has been no comprehensive study on sterol composition of commonly consumed shellfish in the US retail market prior to this work.

The primary source of food composition data in the United States is the USDA Nutrient Database for Standard Reference (SR) (8). SR data are used in conjunction with dietary surveys such as the What We Eat in America component of the National Health and Nutrition Examination Survey (19) and software such as the University of Minnesota Nutrition Data System for Research (20) to estimate dietary intake. The accuracy of these estimates obviously depends on the quality and completeness of the food composition data, including accounting for any variability in the food supply. In 1997, USDA initiated the National Food and Nutrient Analysis Program (NFNAP) (21) to update data in SR using robust representative statistical food sampling plans, validated analytical methods, and comprehensive analytical quality control as discussed in previous communications (22–24). Analytical data from the NFNAP have been used to enhance, replace, or fill in missing values for food components in SR or generate entries for foods not yet represented. Increasing research on the nutritional epidemiology of bioactive non-nutrients has created a need for food composition data on these important components as well (25, 26) in addition to macronutrients, vitamins, and minerals. Therefore, as part of the NFNAP, special interest databases for selected bioactive non-nutrients have been compiled, including choline, flavonoids, fluoride, isoflavones, proanthocyanidins, and carotenoids (see USDA Nutrient Data Laboratory home page, http://www.ars.usda.gov/Services/docs.htm?docid=8964).

Cholesterol values have been included for selected foods in SR since its inception, while data for individual phytosterols were limited prior to SR 14 in in 2001. Food composition data in SR have been revised on an ongoing basis as part of the NFNAP to incorporate analytical values based on statistically representative food sampling plans and validated analytical methods with comprehensive quality control, using the Key Foods approach (27) to prioritize foods and nutrients. As of 1985, all cholesterol values released were based on chemically specific gas chromatographic (GC) analysis, the current standard method of analysis for food composition (Association of Official Analytical Chemists [AOAC]) (28), but until the NFNAP began in 1997, data for many foods were compiled from literature reports and from limited sampling that did not involve a comprehensive, representative, statistical sampling plan and consistent analytical quality control.

Similar to cholesterol, any values for phytosterols in SR prior to the NFNAP were mostly derived from the literature, possibly based on outdated methods and/or limited sampling. Values also included only sitosterol, campesterol, and stigmasterol or ‘total phytosterols’ which was the sum of these three sterols, whereas many foods contain other sterols at significant levels. As part of NFNAP, key foods containing phytosterols were analyzed for these components using current analytical methods to update food composition data, including nuts and seeds (29) and mushrooms (30).

In 2007–2008, six of the most highly consumed crustaceans (shrimp, blue crab, and lobster) and mollusks (clams, scallops, and oysters) were sampled for the NFNAP and analyzed for a range of nutrients to update SR. In addition, phytosterols and sterols unique to marine species were analyzed in these samples. The purpose of this communication is to report on the sterol content of these products occurring in the US market.

## Materials and methods

### Samples

**Fig. 1:**
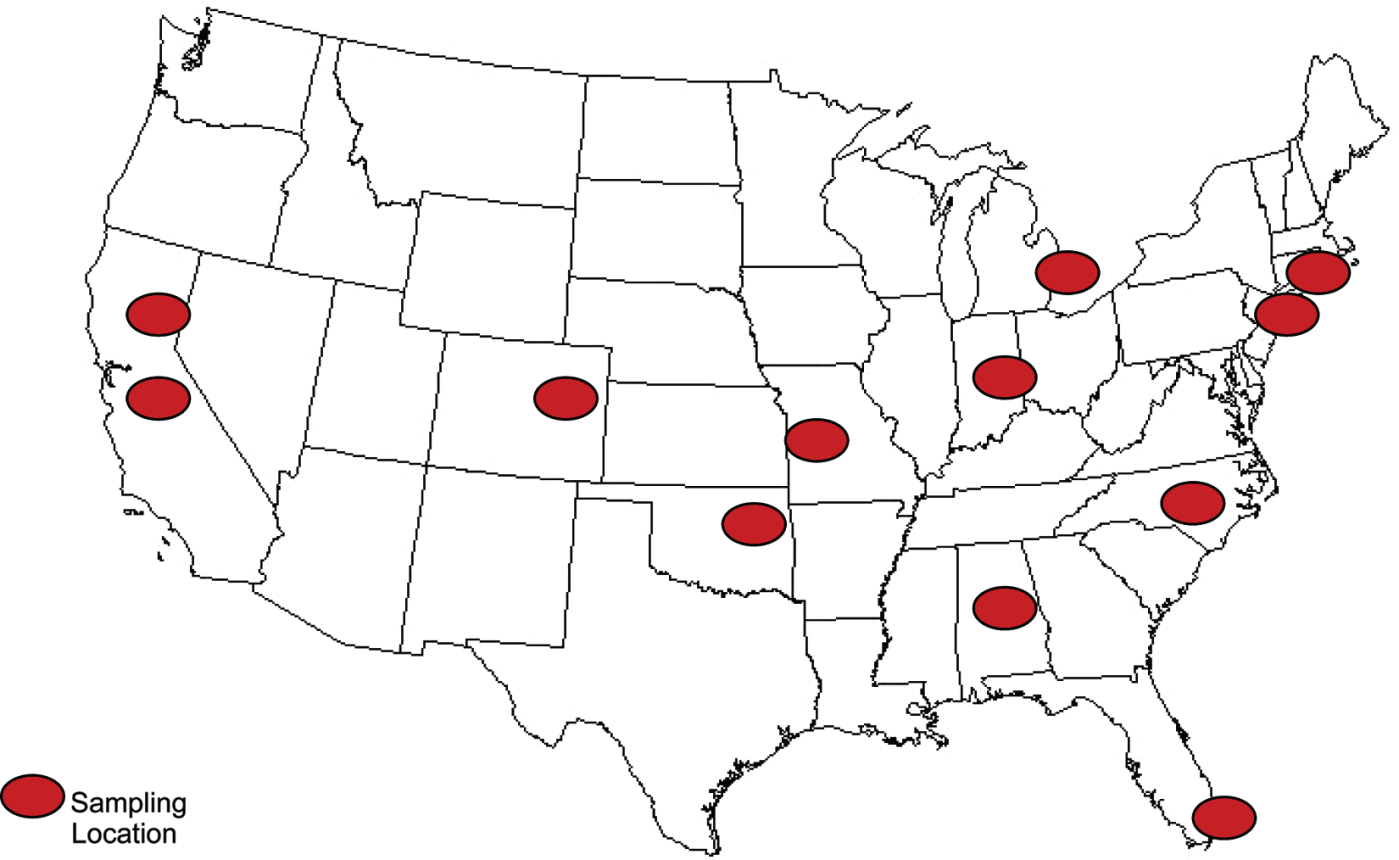
Sampling locations for shellfish in retail markets.

Crustaceans (lobster, blue crab, and shrimp) and mollusks (clams, scallops, and oysters) were selected for analysis based on the US Food and Drug Administration (FDA) top 20 seafoods for voluntary nutrition labeling (31). Canned instead of fresh clams were analyzed because of the ease of finding this form in a nationwide sampling as opposed to finding fresh product. Samples were purchased in February 2007 (shrimp and clams) and May 2008 (oysters, blue crab, lobster and scallops) from one or two retail outlets in each of 12 US cities according to a statistical plan developed for the NFNAP using methodology previously described (22, 23). The sampling plan (Fig. 1) was designed to procure product representative of what is most available in the US retail market; thus, products were not specified to originate from specific producers. Some samples were packaged and some were obtained in bulk from the seafood counter. For some products, the country of origin was available and was documented. The amount of product for each species obtained from each outlet had a range of 0.5–4.5 kg. Additionally, raw clams, oysters, and mussels (2–6 kg each) that had been purchased locally (Blacksburg, VA) from a major supermarket and prepared for another study were assayed to provide some data for products not represented in the national sampling plan.

Samples were procured, packaged, and shipped using methods described elsewhere (32). Prepackaged samples were shipped in their original packages, and bulk seafood was kept in its original wrapping, with each sample placed within a Ziploc^®^ bag, with no more than 2 lb per bag. Samples were frozen for 18–24 h prior to shipping and shipped on dry ice via overnight service to the Food Analysis Laboratory (FALCC) at Virginia Tech (Blacksburg, VA). Canned clams were wrapped in bubble wrap to avoid damage and shipped at ambient temperature via overnight service. Upon receipt, the product labels and visual appearance of the samples were used to verify identity of the products. All samples were kept frozen (−15±3°C) between receipt and preparation. Samples were prepared as described below within 4 weeks of receipt (median: 20 days; range: 6–26 days).

### Sample preparation and compositing

The seafood was obtained steamed (lobster, crab, and oyster) or raw (shrimp and scallops) to give data for nutrients in both raw and, after steaming at Virginia Tech, cooked products. For each species, the sample units from three randomly grouped locations were combined and homogenized to create four triad composites per species. Single-location composites and/or a composite of all locations (national composite) were also prepared for some products. This compositing scheme was one of the alternatives of the overall statistical sampling and analysis plan. In cases where samples were not available at all locations (blue crab, and lobster), composites comprised of four or five locations were prepared. All of the composites were analyzed for a number of other nutrients to update data in SR.

The triad composites were prepared by combining and homogenizing approximately equal weights of the edible portion of individual units, after removal of inedible parts (shells of all species and vein in shrimp) and dissection into pieces of ∼1.25 cm. The pieces were frozen in liquid nitrogen and homogenized using a 6-L stainless steel industrial food processor (Robot Coupe Blixer^®^; Robot Coupe USA, Jackson, MS) while being kept frozen with liquid nitrogen. Aliquots (8–12 g) of each frozen composite were dispensed into 30-mL glass jars with Teflon™-lined lids, covered with aluminum foil, and stored in darkness at −60°C prior to analysis.

A salmon control composite (salmon CC), which was prepared for use as an in-house analytical quality control material, was also included in the analyses. The salmon CC comprised ∼15.8 kg of drained, canned red sockeye salmon, which was homogenized (without liquid nitrogen) using a 30-quart stainless steel industrial food processor (Robot Coupe R30) and distributed among 960 thirty-milliliter glass jars with Teflon-lined lids, under stirring to maintain homogeneity during dispensing. The homogeneity of the salmon CC was validated as described in a previous publication (24).

### Analytical methods

#### Sterol quantification by GC–FID

Reagents and standards were prepared as reported previously (29), and in addition, authentic cholesterol (96.0% purity), 7-dehydrocholesterol (7DHC) (98.0% purity), desmosterol (90.0% purity), and cholecalciferol (99.9% purity) were obtained from Sigma-Aldrich Chemical Co. (St. Louis, MO), and brassicasterol (99.4% purity) was obtained from Steraloids, Inc. (Newport, RI).

Sterols were quantified by capillary gas–liquid chromatography with flame ionization detection (GC–FID) of the trimethyl silyl ether (TMS) derivatives after alkaline saponification of the total lipid extract, with epicholesterol as an internal standard, as described previously (29). Briefly, 20 mL of the total lipid extract (from 80 mL total) of 2 g of sample homogenate (subsampled after thawing for 20 min at 30°C and mixing) was taken for gravimetric determination of total lipid (33), and a 30 mL portion was taken for analysis of sterols by GC–FID. Lipid extracts containing at least 5 mg total lipid were subjected to saponification at 85–89**°**C with potassium hydroxide and 3% ethanolic pyrogallol as an antioxidant, and then the non-saponifiable components were extracted with cyclohexane and derivatized with a 50/50 mixture of pyridine and BSTFA (with 1% TMCS) to form TMS derivatives and injected on the GC. For all analytical components, *β*-sitosterol (corrected for the stated purity) was used for multiple point calibration of a gas chromatograph using epicholesterol as the internal standard. Sterol quantitation in samples was based on individual component/internal standard response ratios applied to the linear regression equation obtained from the instrument calibration performed under identical conditions.

#### GC–MS identification of sterols

Selected samples were also analyzed by gas chromatography–mass spectrometry (GC–MS) to confirm the identity of sterols. The TMS-derivatized extracts containing sterols were analyzed using an Agilent 5890 Series II with 5972 MSD (Agilent Technologies, Santa Clara, CA) and Agilent DB-5 (5% diphenyl/95% dimethyl polysiloxane) GC column (30 m × 0.25 mm × 0.25 µm df) utilizing splitless injection. Carrier gas flow was set at 1 mL/min, and the oven temperature program was 250°C for 2 min, then ramped at 0.5°C /min to 265°C and kept for 25 min for a 57 min total run time. Detector and injector temperatures were both set at 275°C and an 8-min solvent delay was used. Mass spectrometer ionization energy was set at 70 eV, and spectra were scanned in the range of m/z 35–550.

Representative samples of oyster, scallops, crab, lobster, and salmon samples were analyzed. Sterol identification was based on the presence of the expected molecular ion (M+) and/or most of the expected ion fragments, as well as occurrence in the expected GC elution order. The work of Kanazawa (13), Teshima et al. (34), Gordon et al. (35), Rovirosa et al. (36), and Perez et al. (37), Ballantine et al. (38), and Bergquist et al. (39) was used for additional reference. Relative retention times (RRTs) based on the epicholesterol internal standard were established from these results. Subsequently, components in the analytical samples were identified based on the RRT, using GC–MS to confirm closely eluting peaks in individual samples, as necessary. A shorter GC column was used for GC–MS (30 m vs. 60 m used for GC–FID) in order to improve sensitivity for low concentration analytes.

#### Moisture and proximates

Moisture in each composite and in aliquots taken from throughout the dispensing sequence of the salmon CC was determined by vacuum drying to constant weight at 65–70°C and 635 mm Hg. These data were used to verify the homogeneity of the control composite prior to its use (24). Vacuum drying was also used to obtain the dry mass of each sample composite.

### Quality control

Analytical precision was evaluated by analysis of the salmon CC assayed in all batches of samples (*n=*4), a blue crab composite was assayed in two separate batches, and two composites (shrimp and canned clams) was assayed in duplicate or triplicate within a single assay. The salmon CC had previously been analyzed for proximates and cholesterol with other fish and shellfish samples by several analytical laboratories and in parallel with commercially available reference materials having certified values for total lipid and cholesterol to establish validated tolerance limits, as described previously (24). Additionally, NIST SRM^®^ 1546 Meat Homogenate (*n=*2) and NIST SRM^®^ 1845 Whole Egg Powder (*n=*2) (National Institute of Standards and Technology, Gaithersburg, MD) were analyzed to validate the accuracy of cholesterol measurements in this study. NIST SRM^®^ 1566b Freeze-dried Oyster Tissue, which does not have certificate values for sterols, was assayed (*n=*3) to provide information values for the reported sterols in a commercially available sample of similar matrix (it is noted that NIST SRM^®^ 1566b is irradiated, according to the certificate of analysis) (40). There are no reference materials with a similar matrix having certified levels of sterols other than cholesterol. Validation of these analyses relied on previous work (29) and the GC–MS work described earlier to confirm the identity of the components in these matrices.

### Data analysis

Sterol concentrations are reported as mg/100 g fresh composite weight. The results for the freeze-dried oyster tissue (NIST SRM^®^ 1566b) are reported on a dry weight basis (i.e., corrected for residual moisture) to provide reference values for the material and for comparison of the total lipid result to the certified total fat content. The results for NIST SRM^®^ 1566b were estimated on fresh tissue basis assuming moisture content to be equal to the average moisture content of the raw fresh oyster composites.

Means and standard deviations were calculated using Microsoft^®^ Office Excel (Professional Plus edition 2010; Microsoft Corporation, Redmond, WA), and analysis of variance (*α=*0.05) and pairwise comparison of means using the Student Newman-Keuls test with a 95% confidence interval were performed using XLSTAT (version 2011.2.06; Addinsoft, New York, NY). The ratio of the relative standard deviation (RSD) of the measured mean to the expected RSD (HORRAT) for replicate analyses was calculated as described by Horwitz et al. (41). Z-scores for the assayed concentrations of certified components in the reference materials were calculated according to Jorhem et al. (42).

## Results and discussion

### Quality control

Results for total lipid and cholesterol in the standard reference materials and the inter-assay precision for sterols analyzed in reference materials and selected samples are given in Tables 1 and 2. The Z-scores for cholesterol in the reference materials were within the certified range for NIST SRM^®^ 1845 Whole Egg Powder, and the tolerance limits for the salmon control composite and Z-scores were <∣1.0∣, indicating acceptable accuracy (42). The HORRAT for the major sterols was <∣1.5∣ for the blue crab, shrimp, salmon, and NIST SRM^®^ 1566b Freeze-dried Oyster Tissue analyzed in replicate on different days, indicating acceptable precision (41), with a slightly higher but still acceptable HORRAT of 2.3 for cholesterol in blue crab (Table 2).

**Table 1:** Quality control results: accuracy of total lipid and cholesterol analyses in reference materials

Material	Certified range	Assayed mean	*n*	Z-score^a^	HORRAT^b^
Total lipid (g/100 g)					
Salmon control composite^c^	8.37±0.84	8.34	*4*	−0.3	0.57
Cholesterol (mg/100 g)					
Salmon control composite^c^	84.2±5.1	85.5	*4*	1.0	0.37
NIST SRM^®^ 1845 Whole Egg Powder^d^	1864±39	1840	*1*	−0.7	n/a

a Calculated according to Jorhem et al. (42)

b Assayed standard deviation/Expected standard deviation (41)

c Certified mean’ is based on methods described previously (24)

d National Institute of Standards and Technology (Gaithersburg, MD).

**Table 2:** Quality control results: precision of replicate analyses

Composite (*n*)		Total lipid (g/100g)	Occelasterol	24-Norcholesta-5,22-diene-3_-ol	22-Dehydrocholesterol	Dihydrocholesterol	Desmosterol/7-dehydro cholesterol	Brassicasterol	22-Dihydro brassicas terol	24-Methylenecholesterol	Poriferasterol	Clionasterol	Isofucosterol (Δ^5^-avenasterol)	Campesterol	Sitostanol	Campestanol	Unknown Sterol^b^	Cholesterol	Total noncholesterol sterols
Blue Crab	Mean	1.25	0.00	0.23	1.59	<0.2	1.28		0.00	0.93	0.00	0.26	0.00	1.83	0.00	0.00	0.00	97.65	6.22
National composite (2)	SD	0.04	0.00	0.01	0.15		0.02		0.00	0.01	0.00	0.02	0.00	0.02	0.00	0.00	0.00	6.28	0.20
	HORRAT^a^	0.8	–	0.6	1.8	n/a	0.3		–	0.2	–	1.0	–	0.2	–	–	–	2.3	0.8
Salmon	Mean	8.34	0.00	0.65	0.54	0.00	0.39		0.00	0.77	0.28	0.00	0.00	0.00	0.00	0.00	0.00	85.45	2.64
Control Composite (4)	SD	0.14	0.00	0.01	0.00	0.00	0.03		0.00	0.01	0.02	0.00	0.00	0.00	0.00	0.00	0.00	0.90	0.05
	HORRAT	0.6	–	0.3	0.1	–	1.1		–	0.3	0.8	–	–	–	–	–	14.1	0.4	0.4
NIST SRM^®^ 1566b^b^	Mean	11.1	4.35	26.0	38.1	18.7		72.3	5.34	75.8	18.4	40.9	21.8	45.7	0.00	2.74	1.93	234	606
Freeze-dried Oyster Tissue^3^	SD	0.22	0.18	0.92	1.47	0.68		2.45	0.14	2.25	0.65	1.29	0.66	1.32	0.00	0.10	0.08	8.20	20.3
	HORRAT	0.71	0.90	1.02	1.18	0.99		1.14	0.62	1.00	0.96	0.98	0.85	0.91	0.00	0.76	0.80	1.41	1.55
Shrimp (2)	Mean	1.10	0.00	0.36	1.27	0.67	0.48		0.00	0.25	0.32	0.65	0.00	0.96	–	0.00	0.20	123	5.15
	SD	0.05	0.00	0.03	0.08	0.03	0.01		0.00	0.01	0.02	0.03	0.00	0.02	0.00	0.00	0.03	0.28	0.02
	HORRAT	1.1	–	1.4	1.2	0.8	0.3		–	0.5	1.0	0.8	–	0.3	0.00	–	1.8	0.1	0.1
Clams, canned (2)	Mean	1.04	1.75	4.98	8.12	0.61		11.9	0.09	24.9	2.48	4.60	4.78	3.71	–	0.00	0.00	28.3	67.9
	SD	0.01	0.07	0.22	0.34	0.01		0.57	0.13	0.30	0.12	0.22	0.19	0.17	0.00	0.00	0.00	0.07	2.34
	HORRAT	0.2	0.8	1.0	1.0	0.2		1.2	17.5	0.3	1.0	1.1	0.9	1.0	0.00	–	–	0.1	1.2

Note that the certificate of analysis indicates this material was irradiated during preparation.

a Assayed standard deviation/expected standard deviation (41).

b National Institute of Standards and Technology (Gaithersburg, MD) (40).

### GC–MS of sterols

**Fig. 2:**
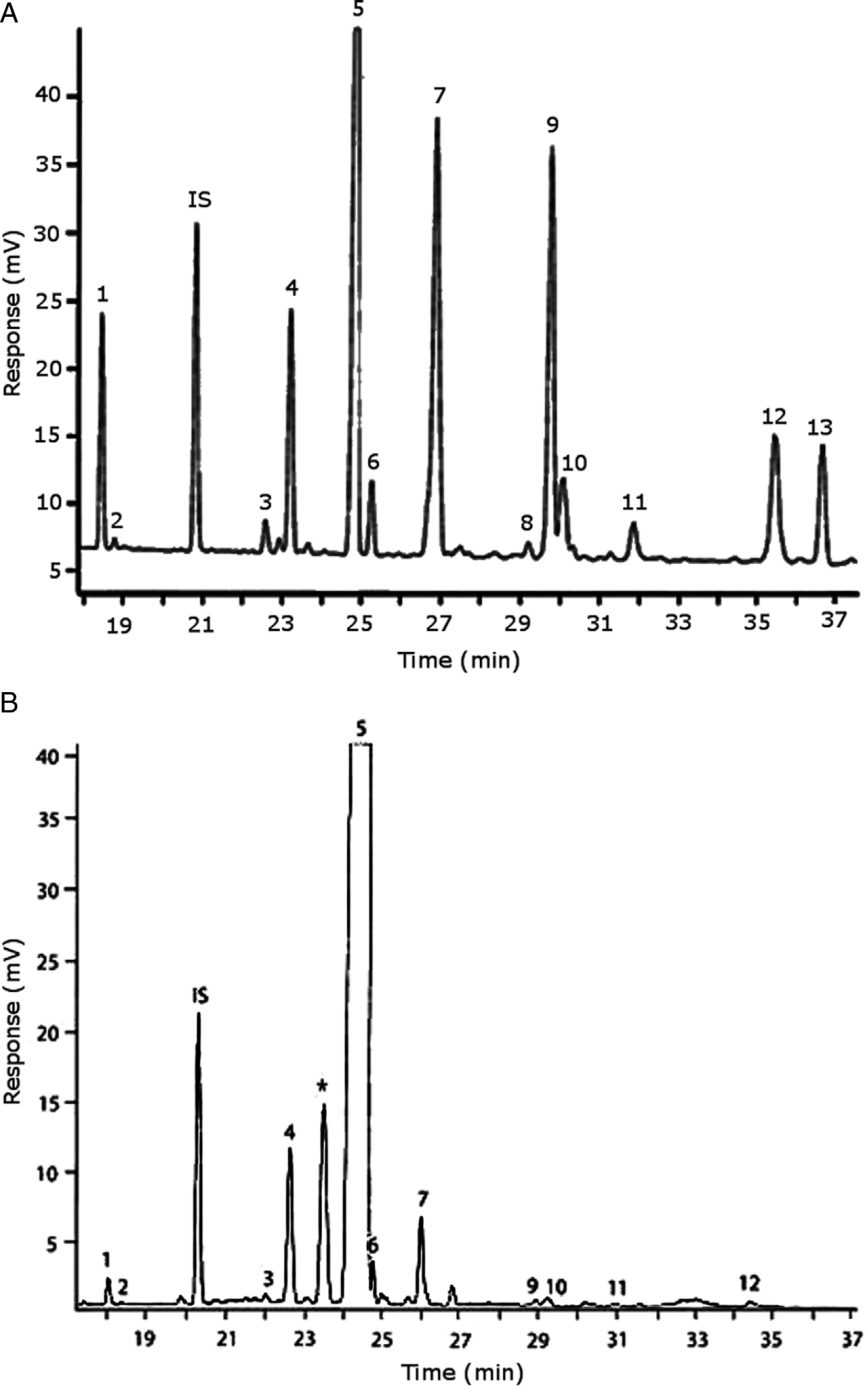
Representative chromatograms for sterols in (A) oyster and (B) lobster. Peak legend: (1) 24-norcholesta-5,22-diene-3β-ol; (2) unknown sterol; (IS) internal standard (epicholesterol); (3) occelasterol; (4) 22-dehydrocholesterol; (5) cholesterol; (6) dihydrocholesterol; (7) brassicasterol in (A) oysters and desmosterol in (B) lobster; (8) 22-dihydrobrassicasterol; (9) 24-methylenecholesterol; (10) campesterol; (11) poriferasterol; (12) clionasterol; (13) isofucosterol (Δ^5^-avenasterol). *Non-sterol (possible α-tocopherol).

Representative GC–FID chromatograms for oyster and lobster are shown in Fig. 2. The oyster and lobster chromatograms were selected to highlight differences between mollusks and crustaceans including the relative amounts of cholesterol and non-cholesterol sterols (NCSs). Table 3 shows the structure of sterols discussed and their common and systematic names. Table 4 summarizes the characteristic mass fragments in GC–MS of the major sterols analyzed. In each case, the molecular ion (M^+^) and most of the expected fragments were found for the TMS-derivatized sterols. Table 4 presents the actual fragments found in selected samples. The sterol 22-dihydrobrassicasterol was likely present in some of the samples but at a concentration too low to obtain a complete GC–MS profile (only the m/z 472 molecular ion was evident) and is therefore not included in Table 4.

**Table 3:** Structure of sterols found in shellfish, and some related sterols

Common name	Systematic name	Formula	Structure
Poriferasterol	Poriferasta-5,22E-dien-3β-ol	C_29_H_48_O	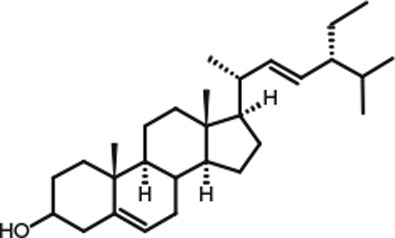
Stigmasterol	Stigmasta-5,22E-dien-3β-ol	C_29_H_48_O	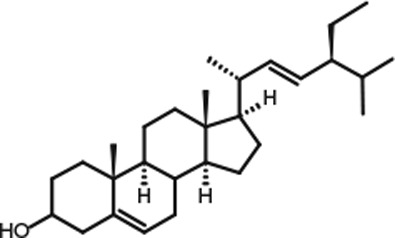
Clionasterol	Poriferast-5-en-3β-ol	C_29_H_50_O	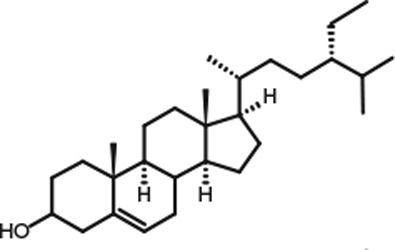
β-Sitosterol (22,23-dihydrostigmasterol)	Stigmast-5-en-3β-ol	C_29_H_50_O	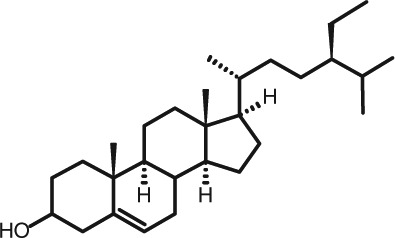
Isofucosterol (▵^5^-avenasterol)	24Z-Ethylidene-cholest-5-en-3β-ol	C_29_H_48_O	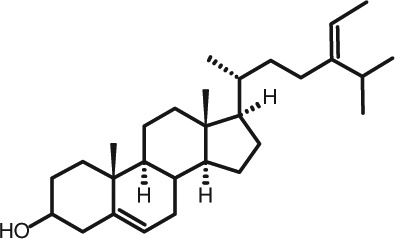
Fucosterol	24E-Ethylidene-cholest-5-en-3β-ol	C_29_H_48_O	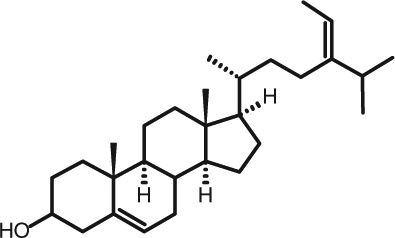
Campesterol	Campest-5-en-3β-ol	C_28_H_48_O	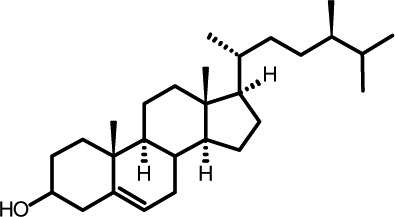
Brassicasterol	Ergosta-5,22E-dien-3β-ol	C_28_H_46_O	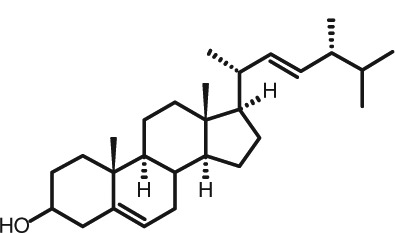
22-Dihydrobrassicasterol	Ergosta-5-en-3β-ol	C_28_H_48_O	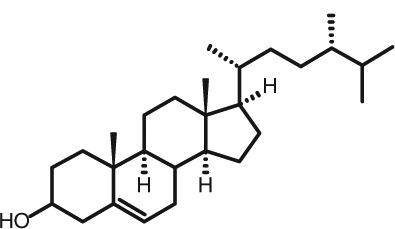
24-Methylene-cholesterol	24-Methylene-cholest-5-en-3β-ol	C_28_H_46_O	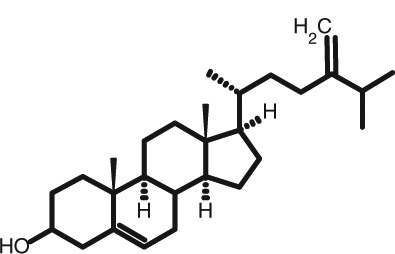
Cholesterol	Cholest-5-en-3β-ol	C_27_H_46_O	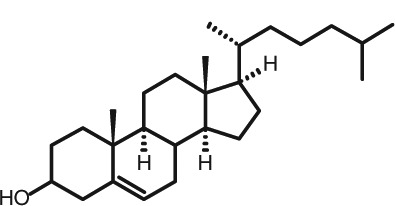
Desmosterol	Cholest-5,24-dien-3β-ol	C_27_H_44_O	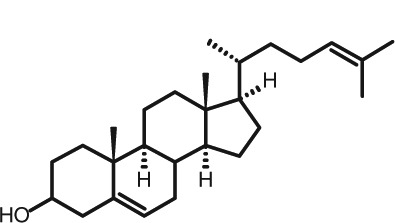
Dihydrocholesterol	Cholestan-3β-ol	C_27_H_48_O	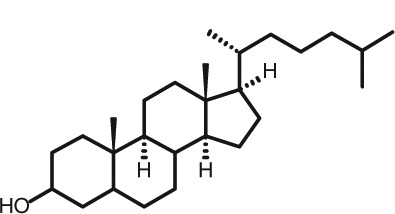
7-Dehydrocholesterol	Cholesta-5,7-dien-3β-ol	C_27_H_44_O	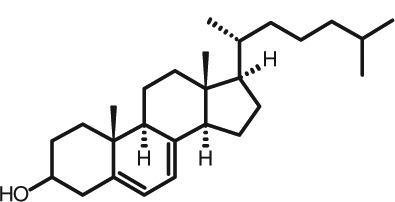
22-Dehydrocholesterol	Cholesta-5,22-dien-3β-ol	C_27_H_44_O	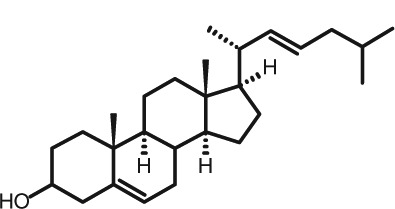
Occelasterol	(22E,24S)-27-Nor-24-methylcholesta-5,22-dien-3β-ol	C_27_H_44_O	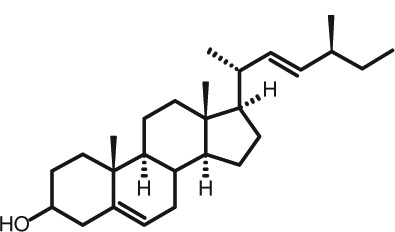
24-Norcholesta-5,22-diene-3β-ol	(22E)-24-Norcholesta-5,22-dien-3β-ol	C_26_H_42_O	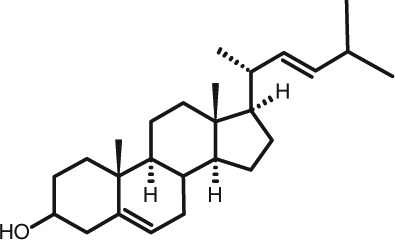

**Table 4:** Characteristic mass fragments for trimethylsilyl (TMS) ether derivatives of sterols in crustaceans and mollusks sample analyzed by gas chromatography–mass spectrometry, corresponding to peaks in the chromatograms in Fig. 2

TMS ether component	Sample	RRT^a^ (min)	M^+^ (m/z)	Other significant fragments^b^ (m/z)
24- norcholesta-5,22-diene-3-ol	Oyster	0.888	442 {5}	427 {1} 352 {8} 337 {4} 313 {9} 255 {13} 215 {6} 213 {4} 129 {53} 97 {100}
Occelasterol	Oyster	1.084	456 {7}	366 {11} 351 {4} 327 {11} 255 {20} 215 {7} 129 {88} 111 {100}
22-Dehydrocholesterol	Oyster	1.115	456 {8}	441{2} 366 {13} 351 {6} 327 {17} 255 {22} 215 {10} 213 {8} 129 {93} 111 {100}
Cholesterol	Oyster	1.198	458 {8}	443 {2} 368 {21} 353 {11} 329 {31} 275 {3} 255 {7} 247 {8} 213 {6} 129 {100}
Dihydrocholesterol	Oyster	1.213	460 {6}	445 {7} 370 {5} 355 {7} 306 {7} 305 {6} 262 {2} 237 {2} 230 {7} 217 {15} 216 {23} 215 {39} 75 {100}
Desmosterol	Lobster	1.279	456 {3}	441 {3} 372 {3} 366 {7} 351 {10} 343 {16} 327 {8} 253 {13} 129 {100}
Brassicasterol	Oyster	1.290	470 {6}	455 {1} 380 {8} 365 {3} 341 {6} 340 {2} 337 {2} 255 {18} 215 {7} 213 {5} 129 {72} 69 {100}
24-Methylenecholesterol	Oyster	1.429	470 {2}	455 {2} 386 {11} 380 {6} 371 {2} 365 {4} 343 {7} 341 {8} 296 {10} 281 {7} 257 {9} 255 {5} 253{8} 213 {5} 129 {100}
Campesterol	Oyster	1.444	472 {7}	457 {2} 382 {18} 267 {8} 343 {24} 289 {2} 261 {5} 255 {8} 213 {6} 129 {100}
Poriferasterol	Oyster	1.527	484 {5}	394 {7} 351 {2} 271 {2} 255 {20} 213 {7} 129 {68} 83 {100}
Clionasterol	Oyster	1.696	486 {6}	471 {2} 396 {16} 381 {7} 357 {20} 303 {2} 275 {5} 255 {7} 213 {7} 129 {100}
Isofucosterol	Oyster	1.754	484 {2}	469 {1} 386 {36} 379 {2} 371 {3} 355 {2} 343{2} 296 {34} 281 {23} 257 {17} 227 {6} 211 {13} 129 {100} 73 {91}

a retention time relative to epicholesterol (internal standard).

b relative peak intensities (percent of base peak) in brackets.

The RRTs for 7DHC, desmosterol, and brassicasterol (which elute in this order) are very close, with potentially overlapping peaks. In all of the mollusks analyzed, GC–MS confirmed brassicasterol as the predominant sterol. In the crustaceans, the mass spectrum of the peaks had fragments, suggesting desmosterol or 7DHC as the primary sterol. In the crustacean samples, the molecular ion for desmosterol/7DHC was larger than the one for brassicasterol, and many more of the expected desmosterol and 7DHC fragments were present, compared to the mass spectrum of the peak at this RRT in the mollusks. These results generally support that desmosterol and/or 7DHC predominates in the crustaceans with only minor, if any contribution of brassicasterol, whereas brassicasterol predominates in the mollusks, with little to no desmosterol or 7DHC. The GC–MS profile of 7DHC reported in the literature (43) is similar to that of desmosterol. However, a couple of the expected GC–MS fragmentation differences between desmosterol and 7DHC (presence of m/z 372 and absence of m/z 325) suggested predominance of desmosterol over 7DHC.

Poriferasterol and stigmasterol have nearly identical RRT and GC–MS profiles, as do clionasterol and sitosterol (44), making it impossible to confirm the identity by GC–MS. Therefore, peak assignments for these sterols were based on literature reports of the isomers occurring in crustaceans and mollusks (13, 34–37). Fucosterol partially coelutes with clionasterol under the analytical conditions employed and may be present in small amounts in some of the seafood samples tested (especially mollusks). This possibility is suggested by a trailing edge shoulder on the clionasterol peak in the GC–FID chromatogram of some mollusk samples and by GC–MS analysis that showed at least two prominent fucosterol ion fragments in the clionasterol profile for oyster and scallops samples.

In many of the shellfish analyzed, a small unknown sterol peak eluting immediately after 24- norcholesta-5,22-diene-3β-ol was present. Although GC–MS analysis was inconclusive due to the very low concentration of this analyte, it matched the RRT of the larger of two peaks found in a GC–FID run of an authentic cholecalciferol standard and was therefore included as a likely sterol-related compound. There was also a relatively large peak in lobster (and other crustaceans) that eluted just before cholesterol (Fig. 2B), which is most likely α-tocopherol based on RRT of an authentic standard, but neither this peak nor the standards appeared in the total ion current chromatogram in the GC–MS analysis under the operating conditions used. Milligram levels of α-tocopherol have been reported in crustaceans (8).

While the GC–MS data and expected elution order generally supported all identifications presented, the possibility of compound coelution and isomeric variation suggests that individual sterol identities should be considered tentative at this point. Further investigation with the aid of NMR could provide additional information to confirm component identities.

### Sterol content of shellfish

Tables 5 and 6 summarize the analyzed concentrations of cholesterol and NCSs in the shellfish composites, in mg/100 g fresh edible weight. Oysters had the highest total sterol content (263 mg/100 g) by a large margin, and scallops and clams had the lowest (94.6 and 98.1 mg/100 g, respectively). There was a clear distinction between the concentration of cholesterol and NCSs in crustaceans (lobster, shrimp, and blue crab) versus mollusks (scallops, clams, oyster, and mussels). Crustaceans had relatively higher cholesterol concentrations [96.2 mg/100 g (blue crab) to 146 mg/100 g (lobster)] and only low levels of NCSs (total <6 mg/100 g). In contrast, mollusks had a lower cholesterol content [23.4 mg/100 g (scallops) to 82.2 mg/100 g (oyster)], while the total concentrations of NCSs were 70.9–181 mg/100 g, two to three times higher than cholesterol (Table 5). The individual NCS detected (Table 6) included the 27-carbon cholesterol metabolites desmosterol, dihydrocholesterol, 7DHC; 24-methylenecholesterol; the 24-methyl and 24-ethyl sterols campesterol, brassicasterol, sitosterol, stigmasterol, fucosterol, isofuctosterol and some of their stereoisomers (dihydrobrassicasterol, clionasterol, poriferasterol); and the 24- and 27-norcholestane derivatives occelasterol and 24-norcholesta-5,22-diene-3β-ol that are unique to marine invertebrates (13).

**Table 5:** Total lipid, moisture (g/100 g), cholesterol, and non-cholesterol sterols (mg/100 g) in crustacean and mollusk composites on fresh weight basis

					Cholesterol (mg/100 g fresh weight)		Total noncholesterol sterols^c^ (mg/100 g fresh)		Total sterols (mg/100 g fresh weight)	
							
Description	NDB number^a^	Composite^b^	Moisture (g/100g)	Total lipid (TL) (g/100g)	Assayed	Mean (± Standard error)	Assayed	Mean (± Standard error)	Assayed	Mean (± Standard error)
Crustaceans										
Blue crab^d^	15139	A	78.67	1.22	109	96.2 ±17.5	6.01	5.65±0.27	115	102±17.8
*Callinectessapidus rathbun*		B	81.32	1.42	61.6		5.11		66.7	
		C	79.09	1.49	118		5.83		124	
Lobster^d^	15144,	A	77.27	1.39	133	146±8.0	4.46	5.03±0.29	137	151±8.2
	15147	B	79.08	1.42	160		5.42		166	
		C	77.98	1.60	144		5.21		150	
Shrimp^e^	15149	A	82.55	1.12	135	129± 3.5	5.48	5.52±0.09	140	134±3.7
*Penaeidaeand Pandalidae*		B	84.57	0.94	123		5.26		128	
		C	84.11	1.10	123		5.15		128	
		D	80.81	1.17	135		6.18		141	
										
Mollusks										
Oyster^d^	15245,	A	76.94	3.60	81.5	82.2± 5.0	183	181±11.0	264	263±15.9
*Crassostrea spp*.	15167,	B	79.77	4.25	74.0		161		235	
	15171	C	77.85	3.57	91.3		199		291	
Scallops^e^	15172	A	81.42	0.850	23.5	23.4± 1.4	75.7	71.2±1.9	99.2	94.6±3.0
*Pectinidae spp*.		B	81.88	0.792	27.3		72.4		100	
		C	85.20	0.738	20.7		66.5		87.2	
		D	81.61	0.773	22.1		70.3		92.4	
Clams^f^	15141	A	79.22	1.02	26.8	29.3± 2.1	62.5	68.8±3.4	89.3	98.1±5.2
*Lamellibranchia spp*.		B	78.50	1.04	28.3		67.9		96.1	
		C	79.15	1.51	34.7		78.6		113	
		D	79.05	1.03	27.5		66.4		93.9	

a Nutrient database entry number from USDA National Nutrient Database for Standard Reference (8);

b each letter represents a composite of samples from three retail outlets in statistical sampling region as described in the text (*Samples*);

c sum of individual sterols in Table 3;

d steamed;

e raw;

f canned.

Occelasterol was found in all mollusks but none of the crustaceans. The sterol 24-norcholesta-5,22-diene-3β-ol was present at 3.5–15 mg/100 g in all mollusks but <0.6 mg/100 g in all crustaceans. Sitostanol and campestanol were not detectable (<0.04 mg/100 g) in any of the crustaceans or in canned clams and were only present at trace levels (<0.165 mg/100 g) in oysters and scallops. Shrimp and all mollusks contained an unidentified sterol, which eluted just after 24-norcholesta-5,22-diene-3β-ol. The low concentration (<1 mg/100 g) was insufficient for GC–MS identification. In crustaceans, 22-dehydrocholesterol was the predominant NCS, comprising 1.11–3.24 mg/100 g (20–60% of NCSs). Interestingly, while campesterol in all shrimp composites and all but one blue crab composite was present at 0.898–2.09 mg/100 g (17–35% of NCSs), in one blue crab and all lobster composites the level was only <0.4 mg/100 g (<7% of NCSs). The blue crab composite with lower levels of 22-dehydrocholesterol and campesterol also had the lowest overall sterol content, including cholesterol (Tables 5 and 6).

**Table 6:** Non-cholesterol sterol content of crustaceans and mollusks (mg/100 g fresh weight). The means are from analysis of the composites listed in Table 5 (*n*=4 for shrimp clams and scallops; *n*=3 for blue crab lobster and oyster)

Description	22-Dehydrocholesterol		Dihydrocholesterol		Desmosterol/7-dehydrocholesterol^a^		Occelasterol		24-Norcholesta-5,22-diene-3β-ol		24-Methylenecholesterol	
					
	Mean	Range	Mean	Range	Mean	Range	Mean	Range	Mean	Range	Mean	Range
Crustaceans												
Blue Crab^e^	1.52	0.707–1.99	0.246	<0.161–0.270	1.41	0.532–3.05	<0.16	<0.161–	0.339	<0.160–0.441	0.703	<0.630–0.741
Shrimp^f^	1.34	1.11–1.54	0.654	0.542–0.860	0.488	0.391–0.635	<0.16	<0.157–	0.452	0.312–0.568	0.212	0.171–0.251
Lobster^e^	2.49	1.91–3.24	0.369	0.354–0.396	1.39	0.991–1.83	<0.16	<0.161–<0.165	0.313	0.269–0.383	0.239	<0.161–0.239
												
Mollusks												
Clams^g^	8.37	7.87–9.47	0.648	0.579–0.801			1.76	1.71–1.83	4.53	3.53–4.98	24.2	23.5–24.9
Oyster^e^	17.8	16.2–18.6	5.47	5.02–6.12			2.42	1.58–3.48	13.3	12.2–15.2	41.9	35.9–49.0
Scallops^f^	10.2	8.10–11.8	1.04	0.805–1.30			3.07	2.58–3.35	5.45	4.51–6.27	16.8	12.9–19.6
Description	Brassicasterol^b^		22-dihydrobrassicasterol		Poriferasterol^c^		Clionasterol^d^		Isofucosterol (▵^5^-avenasterol)		Campesterol	
					
	Mean	Range	Mean	Range	Mean	Range	Mean	Range	Mean	Range	Mean	Range

Crustaceans												
Blue Crab^e^			nd	nd–<0.160	nd	nd–<0.162	0.299	<0.161–0.329	nd	nd–<0.160	1.43	0.286–2.09
Shrimp^f^			nd	nd–0.000	0.35	nd–0.523	0.715	0.435–0.909	nd	nd–nd	1.14	0.898–1.63
Lobster^e^			nd	nd–0.000	nd	nd–<0.165	0.203	<0.165–0.219	nd	nd–nd	0.26	0.174–0.334
												
Mollusks												
Clams^g^	12.65	11.0–15.9	nd	<0.2–	2.47	2.31–2.76	5.48	4.42–8.08	5.06	4.70–5.71	3.60	nd–6.98
Oyster^e^	45.6	39.6–50.8	2.1	1.33–3.50	4.8	3.82–6.00	19.2	16.6–21.3	12.5	11.1–14.0	16.1	9.44–21.0
Scallops^f^	15.9	14.5–19.3	nd	0.000–<0.335	2.6	2.05–3.10	6.56	6.05–7.49	5.70	5.02–6.80	3.99	2.95–6.45

a May contain trace of brassicasterol;

b may contain trace of desmosterol and/or 7-dehydrocholesterol;

c may contain trace of stigmasterol;

d may contain trace of sitosterol;

e steamed;

f raw;

g canned.

Among the mollusk species, the concentrations of NCSs relative to cholesterol were similar although overall, the total NCS was much higher in oysters. The predominant NCS were 24-methylene cholesterol and brassicasterol. The 24-methylene cholesterol concentration differed significantly among scallops, canned clams, and oysters (*P<*0.005), with respective means of 16.8, 24.2, and 41.9 mg/100 g that represented 23–36% of NCSs. The mean brassicasterol concentration was 45.6 mg/100 g (25% of NCSs) in oysters and 12.6–15.9 mg/100 g (18–22% of NCSs) in canned clams and scallops. As discussed above, it was not possible to clearly distinguish the brassicasterol, 7DHC, and desmosterol in these samples, but GC–MS suggested that brassicasterol was the major component of this peak in mollusks (see chromatogram for oysters, Fig. 2A). Other prominent sterols in all mollusks (4–21 mg/100 g) were 22-dehydrocholesterol, isofucosterol, clionasterol, and 24-norcholesta-5,22-diene-3β-ol. Another C-26 sterol with the norcholestane side chain, 22-trans-24-norcholesta-5,7,22-trien-3β-ol or ‘crassosterol’, has been reported in oysters by Teshima and Patterson (45) and others. There may be other ▵^5,7^ sterols present in seafood samples as well, although it has been suggested that some of these are lost due to oxidation (35). Further studies with the aid of NMR would help sort out the complexities of this group of sterols.

### Within-species variability

**Fig. 3:**
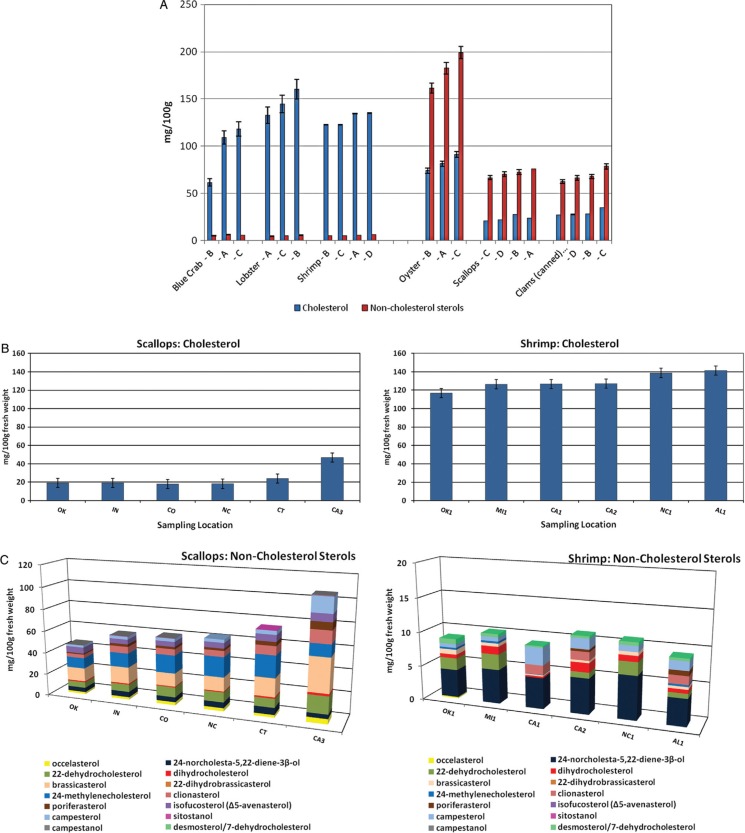
Variability in sterol composition of individual samples of shrimp and scallops (fresh weight basis). Capital letters in panels B and C correspond to the composite in panel A in which the sample was included. Non-cholesterol sterols include all sterols other than cholesterol (cholesterol metabolites, phytosterols, and marine sterols). (A) Composites of crustaceans and mollusks. Each composite contains samples from 3 random locations from statistical sampling regions (Fig. 1). Error bars are the estimated standard deviation based on the relative standard deviation of the same or similar matrix (Table 2). (B) Single-location samples of scallops and shrimp: cholesterol. (C) Single-location samples of scallops and shrimp: non-cholesterol sterols.

Within-species variability was remarkably low in some cases but notably high in others and is illustrated in Fig. 3. In crustaceans, cholesterol ranged from 61.6 to 118 mg/100 g in the three blue crab composites and 161–199 mg/100 g in the three lobster composites. In contrast, the four shrimp composites had remarkably similar cholesterol levels (123–135 mg/100 g). Bragagnolo and Rodriguez-Amaya (46) also found surprisingly little variability in the cholesterol content of farm-raised and different species of wild shrimp (114–139 mg/100 g) and concluded that the origin of samples and size of the shrimp did not have a significant influence on composition. The variability in cholesterol content of lobster and blue crab compared with shrimp is supported by evidence of differences in cholesterol metabolism. Whereas shrimp growth improves with dietary cholesterol supplementation, tissue levels are relatively unaffected (47, 48). On the other hand, cholesterol in lobster and crab has been shown to be influenced by dietary cholesterol (49, 50).

**Fig. 4:**
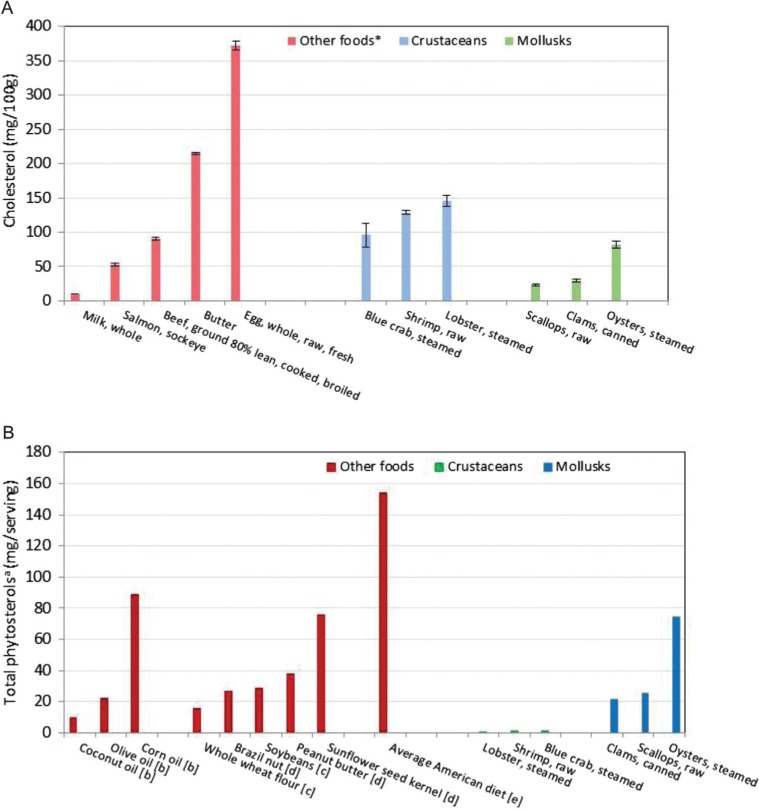
(A) Cholesterol and (B) total phytoserols^a^ in crustaceans and mollusks compared to selected other foods. Error bars in (A) represent ±standard error.*Values for beef, salmon, and egg are from USDA Nutrient Database for Standard Reference (8).^a^Sum of sitosterol, campesterol, stigmasterol, brassicasterol, ▵^5^-avenasterol, sitostanol, campestanol; ^b^Phillips et al. (60), serving size 14g; ^c^Phillips et al. (61), serving size 28g; ^d^Phillips et al. (29), serving size 28g; ^e^Phillips et al. (62), serving size 2000 kcal (8.37 MJ).

**Fig. 5:**
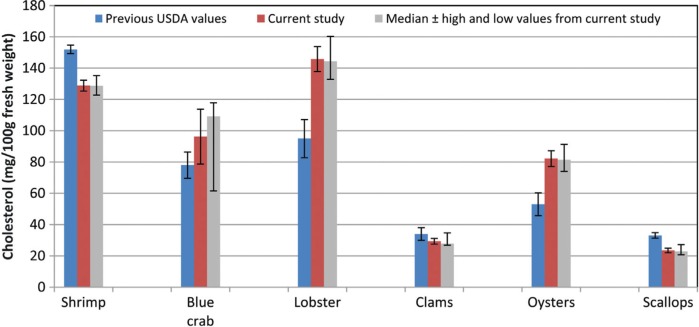
Earlier United States Department of Agriculture data for cholesterol in shellfish (59) versus results from current sampling and analysis. Error bars represent ±2 times the standard error.

In mollusks, the cholesterol and NCS contents varied among the three oyster composites ranging from 74–91.3 and 161–199 mg/,100 g, respectively. Within-species, difference among composites of the other mollusks was <8 mg/100 g for cholesterol in scallops and clams and 9.2–16.1 mg/100 g for NCSs. Each composite included samples from three locations; thus, single-location samples that were part of composites with concentrations at the extreme ends of the range could have had a significantly higher or lower concentration than the composite. Figs. 4B and 4C show the cholesterol and NCS concentrations in six single-location samples of shrimp and scallops that were included in the composites in Fig. 3. For scallops, the NCS among the 3-location composites had a range of only 16.1 mg/100 g, but, composite B (Fig. 3A) contained samples with both the highest and the lowest NCS concentrations of the six single-location samples analyzed (CA3 and OK, Fig. 5C), differing by 60 mg/100 g.

In mollusks, the cholesterol and NCS contents varied among the three oyster composites ranging from 74–91.3 and 161–199 mg/,100 g, respectively. Within-species, difference among composites of the other mollusks was <8 mg/100 g for cholesterol in scallops and clams and 9.2–16.1 mg/100 g for NCSs. Each composite included samples from three locations; thus, single-location samples that were part of composites with concentrations at the extreme ends of the range could have had a significantly higher or lower concentration than the composite. Figs. 4B and 4C show the cholesterol and NCS concentrations in six single-location samples of shrimp and scallops that were included in the composites in Fig. 3. For scallops, the NCS among the 3-location composites had a range of only 16.1 mg/100 g, but, composite B (Fig. 3A) contained samples with both the highest and the lowest NCS concentrations of the six single-location samples analyzed (CA3 and OK, Fig. 5C), differing by 60 mg/100 g.

Kanazawa (14) reviewed the biosynthesis of sterols in marine invertebrates. Among marine sterols, the 24- and 27-norcholestane compounds have been noted as biomarkers for marine diatoms (51–55). The concentration of the two norcholestane compounds, 24-norcholesta-5,22-diene-3β-ol (38) and occelasterol (39), were low in all of the crustaceans (<0.6 mg/100 g), but relatively high (2–15 mg/100 g) in mollusks (Table 6). Shellfish in the retail market may be wild harvested or farmed. Diet and season influence the sterol content of mollusks (14–18, 54), so the variability in composition is likely a reflection of differences in these environmental factors.

## Conclusion

The presence in shellfish of a wide range of sterols, including isomeric forms, makes the analysis and quantitation of sterols in marine species more complex than in animal and plant tissues. The mixture of plant, animal, and unique marine sterols probably arises from the coexistence of sterols derived from the food consumed and from endogenous metabolism in the shellfish. GC–MS was important in identifying some sterols that eluted closely. Thus, literature values based on identification by retention time alone should be questioned in the absence of MS confirmation, especially in the case of brassicasterol, desmosterol, and 7DHC.

American Heart Association guidelines recommend consumption of <300 mg cholesterol per day (55). Based on a serving size of 85 g (3 oz.), clams and scallops would contribute <10% of the daily maximum, while the remaining species would contribute 21% (oyster) to 41% (lobster) of the daily value. In Fig. 5, the cholesterol content per 100 g of the crustaceans and mollusks analyzed is compared to that of some other foods reported in SR (8). While cholesterol in all of the crustaceans and in the oysters is similar to or greater than in the 80% lean cooked beef, total phytosterols in oysters was as high per serving as in some other foods considered to be good sources of phytosterols and to the average diet (Fig. 4B). These levels might be sufficient to impact the effect of the accompanying cholesterol. Ostlund and Lin (56) reviewed the regulation of cholesterol absorption by phytosterols, and Lin et al. (57) showed that a diet containing just 449 mg phytosterols/2000 kcal decreased cholesterol absorption, increased the plasma lathosterol/cholesterol ratio, and increased fecal cholesterol excretion compared to a matched control diet with the same cholesterol content but only 129 mg phytosterols/2000 kcal.

Unlike cholesterol and phytosterols, there has been little research on the impact on CVD risk factors of cholesterol metabolites consumed as part of the diet. Connor and Lin (58) found five NCSs in clams, oysters, and scallops to be at least partially absorbed by the human intestinal mucosa including 22-dehydrocholesterol, brassicasterol, and 24-methylene cholesterol. The other two were identified only as a C-26 sterol and a C-29 sterol. Based on the present work on sterols found in these species, it can be reasonably presumed that these C-26 and C-29 sterols were 24-norcholesta-5,22-diene-3β-ol and clionasterol or ▵^5^-avenasterol (isofucosterol), respectively (Table 3).

The wide variability in the cholesterol content of different samples of blue crab suggests that estimates of dietary cholesterol intake from crab that are calculated using average cholesterol values in food composition databases may not reflect the intake in specific populations consuming the shellfish from one source. Similarly, commonly consumed mollusks and especially oysters have significant levels of NCSs that should be considered. The levels of these sterols varied among samples, with particularly noticeable differences for 24-methylenecholesterol, brassicasterol, and campesterol in oysters. These findings have implications for controlled feeding trials and epidemiological studies involving diets containing shellfish. Although there was remarkably low variability in cholesterol content among lobster and shrimp samples, the potential for between-sample variability seen in blue crab and NCSs in oysters demonstrates the importance of a representative sampling plan to obtaining reliable food composition data and consideration that average sterol composition data for single samples of some species, particularly blue crab and oysters, may not be possible to reliably generalize to the overall market supply.

The detailed sterol composition reported herein provides data beyond cholesterol concentration that may be useful in research on the impact of these commonly consumed shellfish on dietary CVD risk factors.

## References

[1] National Fisheries Institute Top 10 Consumed Seafoods. http://www.aboutseafood.com/about/about-seafood/top-10-consumed-seafoods.

[2] Connor WE, Lin DS (1982). The effect of shellfish in the diet upon the plasma lipid levels in humans. Metabolism.

[3] Childs MT, Dorsett CS, Failor A, Roidt L, Omenn GS (1987). Effect of shellfish consumption on cholesterol absorption in normolipidemic men. Metabolism.

[4] Teupser D, Baber R, Ceglarek U, Scholz M, Illig T, Gieger C (2010). Genetic regulation of serum phytosterol levels and risk of coronary artery disease. Circ Cardiovasc Genet.

[5] Ostlund RE (2002). Phytosterols in human nutrition. Ann Rev Nutr.

[6] Huang J, Frohlich J, Ignaszewski AP (2011). The impact of dietary changes and dietary supplements on lipid profile. Can J Cardiol.

[7] Ling WH, Jones PJ (1995). Dietary phytosterols: a review of metabolism, benefits and side effects. Life Sci.

[8] USDA Agricultural Research Service Beltsville Human Nutrition Research Center Nutrient Data Laboratory. USDA National Nutrient Database for Standard Reference.

[9] Kritchevsky D, Tepper SA, DiTullo NW, Holmes WL (1967). The sterols of seafood. J Food Sci.

[10] Idler DR, Wiseman P (1971). Sterols of crustacea. Int J Biochem.

[11] Teshima S-I, Kanazawa A, Ando T (1972). A C_26_-sterol in the clam *Tapes philippinarum*. Comp Biochem Physiol B: Comp Biochem.

[12] Yasuda S (1973). Sterol compositions of crustaceans—I. Marine and fresh-water decapods. Comp Biochem Physiol B: Comp Biochem.

[13] Kanazawa A (2001). Sterols in marine invertebrates. Fisheries Sci.

[14] Sidwell VD, Loomis AL, Grodner RM (1979). Geographic and monthly variation in composition of oysters *Crassostreavirginica*. Marine Fisheries Rev.

[15] Krzynowek J, Wiggin K, Donahue P (1983). Sterol and fatty acid content in three groups of surf clams (*Spisulasolidissima*): wild clams (60 and 120 mm size) and cultured clams (60 mm size). J Comp Biochem Physiol B: Comp Biochem.

[16] Danton E, Véron B, Mathieu M (1999). Influence of diet level on sterols of diploid and triploid oysters *Crassostreagigas* (Thunberg). J Exp Marine Biol Ecol.

[17] Chikaraishi Y (2006). Carbon and hydrogen isotopic composition of sterols in natural marine brown and red macroalgae and associated shellfish. Organic Geochem.

[18] Tsape K, Sinanoglou VJ, Miniadis-Meimaroglou S (2010). Comparative analysis of the fatty acid and sterol profiles of widely consumed Mediterranean crustacean species. Food Chem.

[19] USDA Agricultural Research Service (ARS) Beltsville Human Nutrition Research Center Food Surveys Research Group (Beltsville MD) and US Department of Health and Human Services Centers for Disease Control and Prevention National Center for Health Statistics (Hyattsville MD). What We Eat in America NHANES 2007–2008.

[20] University of Minnesota Nutrition Coordinating Center Nutrition Data System for Research (NDSR) http://www.ncc.umn.edu/products/ndsr.html.

[21] Haytowitz DB, Pehrsson PR, Holden JM (2007). The National Food and Analysis Program: a decade of progress. J Food Comp Anal.

[22] Pehrsson PR, Haytowitz DB, Holden JM, Perry CR, Beckler DG (2000). USDA's National Food and Nutrient Analysis Program: food sampling. J Food Comp Anal.

[23] Perry CR, Pehrsson PR, Holden JA A revised sampling plan for obtaining food products for nutrient analysis for the USDA national nutrient database. http://www.amstat.org/Sections/Srms/Proceedings/y2003f.html.

[24] Phillips KM, Patterson KY, Rasor AR, Exler J, Haytowitz DM, Holden JM (2006). The role of quality control and reference materials in the National Food and Nutrient Analysis Program. Anal Bioanal Chem.

[25] Coulston AM, Boushey CJ (2008). Nutrition in the prevention and treatment of disease.

[26] Milner JA (2000). Functional foods: the US perspective. Amer J Clin Nutr.

[27] Haytowitz DB, Pehrsson PR, Holden JM (2001). The identification of key foods for food composition research. J Food Comp Anal.

[28] AOAC (2011). Official Methods of Analysis of the Association of Official Analytical Chemists, Current edition. Method 994.10.

[29] Phillips KM, Ruggio DM, Ashraf-Khorassani M (2005). Phytosterol composition of nuts and seeds commonly consumed in the United States. J Agric Food Chem.

[30] Phillips KM, Ruggio DM, Horst RL, Minor B, Simon RR, Feeney MJ (2011). Vitamin D and sterol composition of ten types of mushrooms from retail suppliers in the United States. J Agric Food Chem.

[31] Department of Health and Human Services, US Food and Drug Administration 2006

[32] Trainer D, Pehrsson PR, Haytowitz DB, Holden JM, Phillips KM, Rasor AS (2010). Development of sample handling procedures for foods under USDA's National Food and Nutrient Analysis Program. J Food Comp Anal.

[33] Phillips KM, Ruggio DM, Amanna KR (2008). Extended validation of a simplified extraction and gravimetric determination of total fat to selected foods. J Food Lipids.

[34] Teshima S, Patterson GW, Dutky SR (1980). Sterols of the oyster, *Crassostrea virginica*. Lipids.

[35] Gordon DT, Collins N (1982). Anatomical distribution of sterols in oysters (*Crassostreagigas*). Lipids.

[36] Rovirosa J, Munoz O, San Martin A, Seldes AM, Gros EG (1983). Sterols from the Gorgonian *Lephogorgia subcompressa*. Lipids.

[37] Perez MG, Roccatagliata AJ, Maier MS, Sledes AM, Diaz de Astarloa JM (1996). Main sterols from the Echinoid *Encopeemarginata*. Biochem Syst Ecol.

[38] Ballantine JA, Roberts JC, Morris RJ (1975). Sterols of the Cockle *Cerastodermaedule*: evaluation of thermostable liquid phases for the gas liquid chromatographic–mass spectrometric analysis of trimethylsilyl ethers of marine sterols. J Chrom.

[39] Bergquist PR, Lavis A, Cambie RC (1986). Sterol composition and classification of the Porifera. Biochem Syst Ecol.

[40] NIST Certificate of Analysis—Standard Reference Material^®^ 1566b Oyster Tissue. https://www-s.nist.gov/srmors/certificates/view_certGIF.cfm?certificate=1566B.

[41] Horwitz W, Kamps LR, Boyer DW (1980). Quality assurance in the analysis of foods for trace constituents. J Assoc Off Anal Chem Int.

[42] Jorhem L, Engman J, Schröder T (2001). Evaluation of results derived from the analysis of certified reference materials—a user-friendly approach based on simplicity. Fres J Anal Chem.

[43] Wolf C, Chevy F, Pham J, Kolf-Clauw M, Citadelle D, Mulliez N (1996). Changes in serum sterols of rats treated with 7-dehydrocholesterol-Δ^7^-reductase inhibitors: comparison to levels in humans with Smith-Lemli-Opitz syndrome. J Lipid Res.

[44] Goad LJ, Charlwood BV, Banthorpe DV (1991). Phytosterols. Methods in plant biochemistry.

[45] Teshima S-I, Patterson GW (1981). Δ^5,7^–sterols of the oyster *Crassostrea virginica*. Comp Biochem Physiol.

[46] Bragagnolo N, Rodriguez-Amaya DB (2001). Total lipid cholesterol and fatty acids of farmed freshwater prawn (*Macrobrachium rosenbergii*) and wild marine shrimp (*Penaeus brasiliensis, Penaeus schimitti, Xiphopenaeus kroyeri*). J Food Comp Anal.

[47] Chen H-Y (1993). Requirements of marine shrimp *Penaeusmonodon* juveniles for phosphatidylcholine and cholesterol. Aquaculture.

[48] Morris TC, Samocha TM, Davis DA, Fox JM (2011). Cholesterol supplements for *Litopenaeusvannamei* reared on plant based diets in the presence of natural productivity. Aquaculture.

[49] D'Abramo LR, Baum NA, Bordner CE, Conklin DE, Chang ES (1985). Diet-dependent cholesterol transport in the American lobster. J Exp Marine Biol Ecol.

[50] Van den Oord A (1964). The absence of cholesterol synthesis in the crab, *Cancer pagurus* L. Comp Biochem Physiol.

[51] Giner J-L, Wikfors GH (2011). Dinoflagellate sterols’ in marine diatoms. Phytochem.

[52] Leblond JD, Chapman PJ (2002). A survey of the sterol composition of the marine dinoflagellates *Kareniabrevis Kareniamikimotoi* and *Karlodiniummicrum*: distribution of sterols within other members of the class Dinophyceae. J Phycol.

[53] Moldowan JM, Jacobson SR (2000). Chemical signals for early evolution of major taxa: biosignatures and taxon-specific biomarkers. Int Geology Rev.

[54] Krishnamoorthy RV, Lakshmi GJ, Biesiot P, Venkataramiah A (1980). Seasonal variations in sterol content of the oyster *Crassostreavirginica* (Gmelin) from natural reefs in the Mississippi sound. Indian J Marine Sci.

[55] Lichenstein AH, Appel LJ, Brands M, Carnethon M, Daniels S, Franch HA (2006). Diet and lifestyle recommendations revision 2006: a scientific statement from the American Heart Association Scientific Committee. Circulation.

[56] Ostlund RE, Lin X (2006). Regulation of cholesterol absorption by phytosterols. Curr Atheroscler Rep.

[57] Lin X, Racette SB, Lefevre M, Spearie CA, Most M, Ma L (2010). The effects of phytosterols present in natural food matrices on cholesterol metabolism and LDL-cholesterol: a controlled feeding trial. Eur J Clin Nutr.

[58] Connor WE, Lin DS (1981). Absorption and transport of shellfish sterols in human subjects. Gastroenterol.

[59] USDA, Human Nutrition Information Service (1987). Composition of foods: finfish and shellfish products; raw, processed, prepared. Agriculture handbook No. 8–15.

[60] Phillips KM, Ruggio DM, Toivo J (2002). Free and esterified sterol composition of edible oils and fats. J Food Comp Anal.

[61] Phillips KM, Ruggio DM, Ashraf-Khorassani M (2005b). Analysis of steryl glucosides in foods and dietary supplements by solid-phase extraction and gas chromatography. J Food Lipids.

[62] Phillips KM, Tarrago-Trani MT, Amanna KR (1999). Phytosterol content of experimental diets differing in fatty acid composition. Food Chem.

